# Editorial: Harmonization strategies and considerations in neuroimaging studies

**DOI:** 10.3389/fneur.2023.1165263

**Published:** 2023-03-07

**Authors:** Emily L. Dennis, Ilya M. Veer, Maxime Descoteaux, David F. Tate, Elisabeth A. Wilde

**Affiliations:** ^1^Department of Neurology, The University of Utah, Salt Lake City, UT, United States; ^2^Developmental Psychology, University of Amsterdam, Amsterdam, Netherlands; ^3^Department of Computer Science, Université de Sherbrooke, Sherbrooke, QC, Canada

**Keywords:** harmonization, neuroimaging, big data, editorial, multi-site

As the body of human neuroimaging literature has expanded, so too has our understanding of best practices regarding neuroimaging analyses. Analyses of complex behavioral phenotypes to date have generally been underpowered, with a median sample size of 25, while thousands are needed for replicable results (1). The necessary sample size for simpler phenotypes is smaller, but still beyond what many individual labs can feasibly collect. To address this, cross-site harmonization has emerged, both prospective for coordinated multi-site studies, and retrospective for combining existing or legacy data with different acquisition parameters. The number and size of multi-site studies using coordinated imaging acquisition protocols have grown, including the Adolescent Brain Cognitive Development Study [ABCD, (2)], the Alzheimer's Disease Neuroimaging Initiative [ADNI, (3)], and the Transforming Research and Clinical Knowledge in TBI [TRACK-TBI, (4)], among others.

There are six papers in this Research Topic: two overviews of the approaches, considerations, and caveats of cross-site harmonization, two applications, one extension of a common tool to address data-sharing issues, and one overview of a new prospectively harmonized MRI study in Italy. We start the Research Topic with an overview of the various techniques for retrospective harmonization. Bayer et al. provide a guide for researchers looking to apply harmonization techniques to their work, including linear mixed effects models, several variants of ComBat, adjustments based on image quality metrics, normative modeling, and deep learning approaches. This exhaustive review offers an excellent overview of the strengths and weaknesses of each of these approaches, as well as of their statistical foundations, ease of use, including software options, and suitability depending on data type, context, and the specific research question.

Harris et al. present an overview of the specific considerations in harmonizing magnetic resonance spectroscopy (MRS) data. The ability of MRS to measure brain metabolites *in vivo* is of particular interest to many clinical researchers who investigate the impact of various clinical conditions and/or treatments on the metabolic milieu of the brain. This review identifies common variables associated with MRS data collection that limit the generalizability and reproducibility of MRS research findings and that might be amenable to a growing number of potential harmonization efforts. This review suggests various strategies that can be applied to both prospective and retrospective data at various levels of data acquisition or processing. After reading this review, readers should understand the variables that might affect MRS harmonization, some strategies to deal with these variables, and the value and importance of pushing this science forward in the clinical research application of MRS.

The next four papers in the Research Topic focus on ComBat, one of the techniques discussed by Bayer et al. and Onicas et al. apply ComBat to harmonize structural connectivity metrics across a multi-site study of pediatric concussion. Data from six sites were collected with prospective harmonization of protocols. Harmonization effects were compared between two processing stages at which ComBat could be applied: (1) on the weighted adjacency matrices based on regional fractional anisotropy (matrix harmonization), and (2) on the global network metrics derived from the weighted adjacency matrices (parameter harmonization). The authors demonstrate robust confounding effects of scanner type when ComBat is not applied to the multi-site data. NeuroComBat was used for harmonization. Parameter harmonization removed more site effects than matrix harmonization, but both preserved expected biological associations. The authors conclude that parameter harmonization preserves more of the true network topology, while also being less computationally intense.

To address concerns related to potential bias resulting from scanner-related differences in volumetric analysis, Treit et al. combine non–harmonized T1–weighted data from three MRI centers with different acquisition protocols. First, human traveling phantoms were used to quantify within–participant brain volume variability and then calculate site-specific correction factors to address these differences. Additionally, cross-sectional lifespan volume trajectories were examined in healthy participants scanned at these sites across the age spectrum. Trajectories of volume vs. age were then compared before and after the application of traveling phantom-based site-specific correction factors, as well as correction using the ComBat open–source method. Although small systematic differences between sites were observed in the traveling phantom analysis, correction for site using either method had little impact on the lifespan trajectories, suggesting that age–related changes may outweigh systematic differences between scanners for volumetric analysis.

Bostami et al. present an extension of ComBat that allows for cross-site harmonization without data sharing. Federated learning is a relatively new concept but has great potential for less cumbersome data combinations from sites around the world while reducing concerns about privacy and resource demands attributable to sharing. The goal for Combat-DC is that local sites make their data available to an aggregator node, which determines site effects based on data distributions and then returns harmonized parameters to the local site. In doing so, it does not require that raw data are shared. Bostami et al. demonstrate that Combat-DC effectively reduces site effects across a range of data distributions, permitting reliable between-site combination of functional imaging data.

Lastly, Nigri et al. provide an overview of a new prospectively harmonized MRI study in Italy, the RIN-Neuroimaging Network, which is a national consortium dedicated to identifying subject-specific *in-vivo* neuroimaging biomarkers of diverse neurological and neuropsychiatric conditions. RIN regroups 23 Italian Scientific Institutes of Hospitalization and Care (IRCCS) with technological and clinical specializations in the neurological and neuroimaging field. The paper provides a description of (1) guidelines for a minimum standard clinical qualitative MRI assessment for the main neurological diseases, (2) standard operating procedures (SOPs) for quality control in preclinical acquisition on harmonization phantoms, (3) SOPs for clinical acquisition and advanced harmonized quantitative MRI protocols for healthy human participants, and lastly, (4) large-scale multimodal data collection, archiving, and sharing of patient and animal model datasets.

Together, this Research Topic of papers highlights the challenges and complexities of multi-site neuroimaging data collection and aggregation and emerging strategies for the harmonization of these data. Harmonization is a critical step in extending the impact of past and future neuroimaging analyses and replicating discovery, so identifying methods and best practices is crucial to advance research and translate findings to clinical practice.

## Author contributions

All authors listed have made a substantial, direct, and intellectual contribution to the work and approved it for publication.
